# Heparin-Immobilized Polyethersulfone for Hemocompatibility Enhancement of Dialysis Membrane: In Situ Synchrotron Imaging, Experimental, and Ex Vivo Studies

**DOI:** 10.3390/membranes13080718

**Published:** 2023-08-03

**Authors:** Denis Kalugin, Jumanah Bahig, Ahmed Shoker, Amira Abdelrasoul

**Affiliations:** 1 Department of Chemical and Biological Engineering, University of Saskatchewan, 57 Campus Drive, Saskatoon, SK S7N 5A9, Canada; shotoden@gmail.com; 2 Division of Biomedical Engineering, University of Saskatchewan, 57 Campus Drive, Saskatoon, SK S7N 5A9, Canada; qjb825@mail.usask.ca; 3 Kinesiology, University of Saskatchewan, 87 Campus Drive, Saskatoon, SK S7N 5B, Canada; 4 Nephrology Division, College of Medicine, University of Saskatchewan, 107 Wiggins Rd., Saskatoon, SK S7N 5E5, Canada; ahmed.shoker@usask.ca; 5 Saskatchewan Transplant Program, St. Paul’s Hospital, 1702 20th Street West, Saskatoon, SK S7M 0Z9, Canada

**Keywords:** heparin, pseudo-zwitterionic, synchrotron SR-μCT, hemocompatibility, hemodialysis membrane

## Abstract

The goal of the current study is to enhance the hemocompatibility of polyethersulfone (PES) membranes using heparin immobilization. Heparin was immobilized covalently and via electrostatic interaction with the positively charged PES surface (pseudo-zwitterionic (pZW) complex) to investigate the influence of each method on the membrane hemocompatibility. In situ synchrotron radiation micro-computed tomography (SR-µCT) imaging, available at the Canadian Light Source (CLS), was used to critically assess the fibrinogen adsorption to the newly synthesized membranes qualitatively and quantitatively using an innovative synchrotron-based X-ray tomography technique. The surface roughness of the synthesized membranes was tested using atomic force microscopy (AFM) analysis. The membrane hemocompatibility was examined through the ex vivo clinical interaction of the membranes with patients’ blood to investigate the released inflammatory biomarkers (C5a, IL-1α, IL-1β, IL-6, vWF, and C5b-9). The presence and quantitative analysis of a stable hydration layer were assessed with DSC analysis. Surface modification resulted in reduced surface roughness of the heparin-PES membrane. Both types of heparin immobilization on the PES membrane surface resulted in a decrease in the absolute membrane surface charge from −60 mV (unmodified PES) to −13 mV for the pZW complex and −9.16 mV for the covalently attached heparin, respectively. The loss of human serum fibrinogen (FB) was investigated using UV analysis. The PES membrane modified with the heparin pseudo-ZW complex showed increased FB retention (90.5%), while the unmodified PES membrane and the heparin covalently attached PES membrane exhibited approximately the same level of FB retention (81.3% and 79.8%, respectively). A DSC analysis revealed an improvement in the content of the hydration layer (32% of non-freezable water) for the heparin-coated membranes compared to the unmodified PES membrane (2.84%). An SR-µCT analysis showed that the method of heparin immobilization significantly affects FB adsorption distribution across the membrane thickness. A quantitative analysis using SR-µCT showed that when heparin is attached covalently, FB tends to be deposited inside the membrane pores at the top (layer index 0–40) membrane regions, although its content peak distribution shifted to the membrane surface, whereas the unmodified PES membrane holds 90% of FB in the middle (layer index 40–60) of the membrane. The ex vivo hemocompatibility study indicates an improvement in reducing the von Willebrand factor (vWF) for the heparin pseudo-ZW PES membrane compared to the covalently attached heparin and the untreated PES.

## 1. Introduction

Hemodialysis (HD) is a life-saving procedure for patients which kidney function below 10% to 15%, resulting in the kidneys’ inability to filter the blood and produce urine. Each year, over 2 million people worldwide go through this essential procedure; however, this number is estimated to be only around 10% of all people who need this life-saving procedure [1]. Even those who are able to clean their blood in HD sessions experience some HD-related complications and health problems due to blood clotting.

Blood clotting and related health problems after HD sessions are believed to be associated with protein adsorption. The adsorbed proteins provoke a coagulation cascade and complement the activation and adhesion of red blood cells and further fibrosis [2], which causes severe health problems for HD patients and even death [3,4,5]. Although many research studies are devoted to studying the mechanisms of protein adsorption onto different surfaces, the membrane fouling that occurs during HD sessions is still not well explained due to the highly heterogeneous composition of blood. Our research group focuses on in-depth assessment of the protein adsorption that occurs during HD, as well as revealing the key factors that affects human serum protein adsorption on clinical HD modules that are currently used in Canadian hospitals [6,7,8]. We also used synchrotron imaging to investigate in detail the main human serum protein adsorption across HD membrane thickness [6,8,9,10], which is important for understanding the protein adsorption behavior and for further development of a controlled HD process without severe consequences for patients’ health.

Heparin is naturally occurring glycosaminoglycan, i.e., a long, linear polysaccharide consisting of repeating disaccharide units, that has been widely used as an anticoagulant reagent since the 1940s due to its ability to interact with coagulation factors XIa, IXa, Xa, and IIa (thrombin). The use of heparin for hemodialysis (HD) requires the injection of a heparin solution in the patient’s blood [11,12,13] or/and using heparin-covered HD membranes and other parts of dialysis equipment [14,15,16]. Independently of the use method, severe health problems may arise due to with the use of heparin, such as heparin-induced thrombocytopenia (HIT) [17,18,19,20,21,22], hypertriglyceridemia [23], anaphylaxis [24], bone mineral disease [25], hyperkalemia [26], catheter-related sepsis [27], skin necrosis [28], etc. The most severe side effect is HIT, which results is blood clotting [19,29]. In the more severe, potentially life-threatening form, type II HIT heparin exposure induces both bleeding and thromboembolic complications; thus, 73% (the number of HD sessions is 120) of HD sessions were reported to result in multiple fibers clotting (red or rose-colored dialyzer) (Grade 3) or clotting of the dialyzer (Grade 4) when a heparin-coated HD membrane module was used [29].

One of the main causes for these side effects of using heparin is believed to be associated with the surface negative charge that arises when heparin is applied for a covering HD membrane surface [30], since heparin contains sulfonic and carboxylic groups that are able to carry a negative charge. The negatively charged surfaces are believed to be associated with the increased adsorption of proteins and overall fouling that can be considered as the first step to complement activation with further fibrosis [30]. The presence of heparin is intended to reduce this process or even to prevent it, but in vivo HD sessions indicate that heparin does not guarantee the exclusion of blood clotting and related health problems [17,19,20,21,22,24,26,28,31,32,33,34,35,36,37,38].

The trend of latest decade in developing hemocompatible materials for membrane applications is to cover polymer surfaces with near-zero charged structures, which carry equal amount of positive and negative charges (zwitterionic molecules). The presence of charged moieties results in the appearance of a hydration layer that creates a strong barrier against foulant adsorption, and a total near-zero charge prevents adsorption via an electrostatic interaction [39]. This approach was successfully implemented for creating low-fouling and low-fibrosis materials [40,41,42,43,44].

In the present study, we immobilized heparin via covalent and ionic interactions to investigate the influence of the immobilization method on the membrane properties, performance, and biocompatibility. The main objectives of this study are outlined as follows: (i) investigate the influence of the heparin immobilization method on the membrane morphology and surface charge; (ii) evaluate the change in FB adsorption and its distribution across the membrane thickness using an advanced in situ SR-µCT innovative technique; (iii) assess the FB loss as vital human serum protein through synthesized membranes compared to the current clinical membrane; (iv) investigate water stability and non-freezable water around the heparin-coated membrane; and (v) perform in vitro clinical tests to estimate the influence of the heparin immobilization method on the inflammatory biomarkers released in HD patients’ blood.

## 2. Materials and Methods

### 2.1. Materials and Reagents

The PES membrane was supplied by Sterlitech, Auburn, WA, USA. Human serum fibrinogen (FB); heparin sodium salt from porcine intestinal mucosa (H3149-10KU, Grade I-A, ≥180USP); diethylene triamine (DETA); and glutaraldehyde (25% water solution) were purchased from Sigma-Aldrich, Oakville, ON, Canada. The saline solution was provided by St. Paul Hospital. Gold nanoparticles were provided by Nanopartz™, Barberry Pl, Loveland, OH, USA. These nanoparticles were conjugated to human proteins (albumin, fibrinogen, and transferrin) to be detected in the SR-μCT.

#### 2.1.1. PES Membrane Surface Modifications

##### PES Membrane Surface Modification with NH_2_ Groups

The amino groups were directly attached to the PES surface via aminolysis [45]. The PES membrane was placed into a 50% water DETA solution for 3 h, followed by further washing with deionized water several times. The membranes were immediately used for next step of the surface modification.

##### PES Membrane Modification with Heparin–Pseudo-ZW Complex

The PES membrane with NH_2_ surface groups was placed in a water solution of heparin (1 mg/mL) and left overnight at +4 °C. The resultant membrane was washed with deionized water several times and stored in deionized water before use.

##### PES Membrane Modification with Covalently Attached Heparin

The PES membrane with NH_2_ surface groups was treated with glutaraldehyde (2% water solution) for 1 h, followed by further washing with deionized water. Then, the membrane was immediately used for the next step, in which the membrane was placed in a heparin solution (1 mg/mL) and left overnight at +4 °C. The resultant membrane was washed with deionized water several times and stored in deionized water before use.

#### 2.1.2. Synchrotron Imaging at Canadian Light Source (CLS)

**Figure 1:**
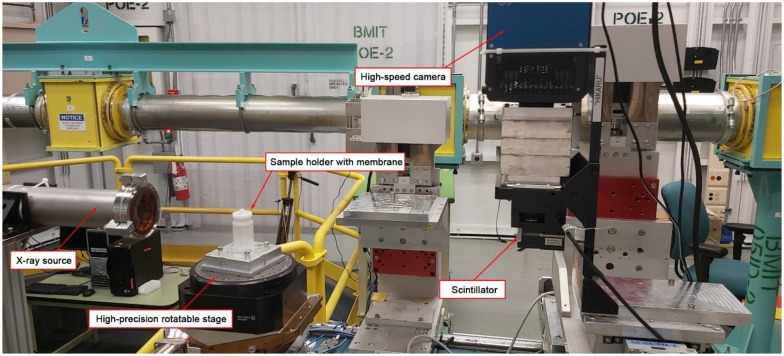
Photo of the experimental SR-μCT setup at the 05B1-1 beamline at the Canadian Light Source.

This X-ray microtomographic study was conducted at the 05B1-1 beamline located at the Canadian Light Source (CLS) facility, Saskatoon, Canada, which is shown in Figure 1. A filtered white beam, used to visualize protein adsorbed onto and inside a membrane surface, was detected using beam monitor AA-40 (500 μm LuAG scintillator, Hamamatsu, Japan) coupled with a high-resolution camera PCO Dimax HS (PCO, Kelheim Bavaria, Germany), with a field of view (FOV) of 4.4 × 2.2 mm, and pixels of 5.5 µm.

**Figure 2:**
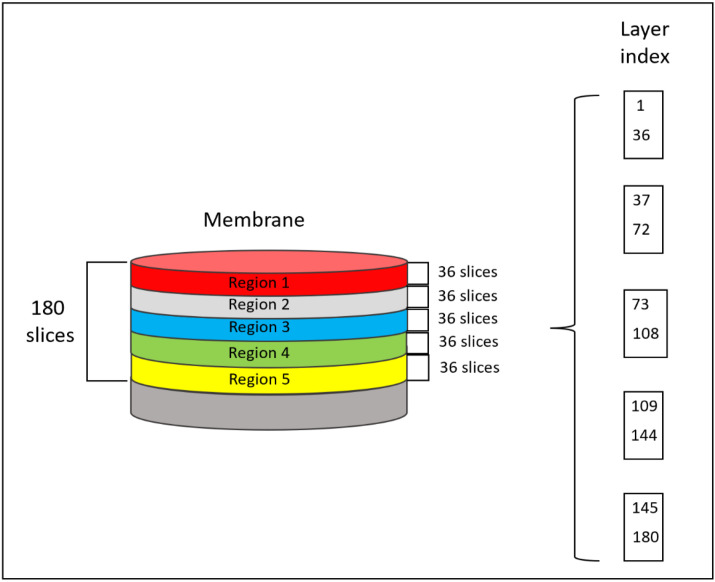
Illustration of layer arrangement when performing SR-μCT layer-by-layer analysis.

The SR-μCT is performed in such a way that the membrane thickness is “sliced” by the X-ray beam into many layers and each layer can be analyzed separately. The layer count starts from the membrane top surface and the layer increase coincides with the solution flow direction (see Figure 2).

The membrane thickness was modeled by 5 regions of interest (ROI), as is shown in Figure 2. Region 1 represents the very top membrane surface. The bottom membrane parts are located in Region 5. Each of the three regions contain 40 layers of the membrane thickness; therefore, the flow direction of the protein solution coincides with the direction of increase of the layer index within the membrane cross-section.

Customized gold nanoparticles, purchased from Nanopartz™, were conjugated with FB for detection during the SR-μCT analysis, since gold has a high attenuation coefficient and X-ray adsorption, thus improving the images’ resolution and quality. Then, the resultant collected radiographs were converted into graphical images and analyzed as described in our recent papers [6]. The CT reconstruction was performed utilizing open-source software Ultra-Fast-Online (UFO) package available at CLS [46]. The converted images were quantitatively treated with open-source ImageJ software (Fiji, GPLv3+) to calculate the amount of adsorbed FB on each membrane level.

**Figure 3:**
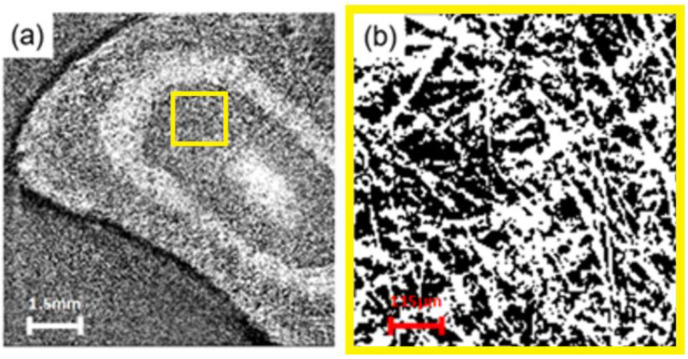
(**a**) Raw image of CT scans and (**b**) zoomed image of selected area. Adapted from [47].

The obtained frames are gray and black with no clear features, as shown in Figure 3a. Following the enhancement of the brightness, adjustment of the threshold, conversion to mask and choosing of the colors, the characteristics could be clearly observed. In the computer tomography (CT) scan, shown in Figure 3b, different materials can be identified depending on their X-ray absorption properties. Materials with high extinction coefficients, which have strong absorption properties, appear as bright areas. In order to ensure the accuracy of the data, four measurements were carried out for each sample. The data presented in the discussion are an average of the measurements. 

#### 2.1.3. In Vitro Investigation of FB Depletion and FB Adsorption

An in vitro ultrafiltration of human serum protein was conducted to ensure the ability of the membrane to retain vital proteins. The In vitro investigation was conducted by filtering aqueous solutions of a model protein (fibrinogen) so as to simulate the patient’s blood. The concentration of proteins simulated the average concentration of the proteins in male and female bodies, i.e., FB had a concentration of 2 mg/mL. The detailed procedure and the experimental setup have been published in our recent study [9]. The concentration of FB was measured using UV–visible at room temperature. The thickness of the FB layer on the membrane surface was measured using AFM.

#### 2.1.4. Analytical Techniques

##### X-ray Photoelectron Spectroscopy (XPS) Analysis

All the X-ray photoelectron spectroscopy (XPS) measurements were collected using a Kratos (Manchester, UK) AXIS Supra system at the Saskatchewan Structural Sciences Centre (SSSC) under UHV conditions. This system is equipped with a 500 mm Rowland circle monochromated Al K-α (1486.6 eV) source and combined hemi-spherical analyzer (HSA) and spherical mirror analyzer (SMA). A spot size of 300 × 700 microns was used. All the survey scan spectra were collected in the −5–1200 binding energy range in 1 eV steps with a pass energy of 160 eV. High resolution scans of 4 regions were also conducted using 0.1 eV steps with a pass energy of 20 eV. An accelerating voltage of 15 keV and an emission current of 10 mA were used for the analysis. The resultant spectra were analyzed using CasaXPS software (CasaXPS Version 2.3.25) [48].

##### Atomic Force Microscopy (AFM) Analysis

An AFM analysis of the PES membranes surface was performed using an NGauge AFM Microscope (Model 1.1). The analysis was performed on 1 × 1 cm samples (scanning area 10 × 10 μm) that were attached to the metal support surface with the help of double-sided carbon tape to minimize the surface charge, which affects the scanning procedure. The resultant AFM images were postprocessed in Gwyddion software (GNU General Public License version 2.0 (GPLv2)). AFM was used to assess the unmodified and modified membrane roughness, in addition to the FB thickness on the membrane surfaces.

##### UV–Visible Spectrometer Analysis

A UV analysis was performed using an Ocean Optics UV–Visible spectrometer, which is equipped with a deuterium tungsten light source, providing spectral measurements in the range of 200–1100 nm. The analyzed protein solution samples (about 1.5 mL) were put in a 1 × 1 cm quartz cuvette with further collection of the UV–vis spectra. For quantitative information about the protein content, the optical density at a 280 nm wavelength was analyzed using OceanView software (version 1.4.1 licensed) (Six solution samples with known protein concentrations were used for preparing the calibration curve. A saline solution was used for background subtraction to improve the quality of the protein content calculations. 

##### Surface Charge Measurement

Surface charge measurements of the membranes were performed with the use of a zeta potential analyzer (Zetasizer-Nano Series, Malvern Instruments Ltd., Malvern, UK, ±0.01 mV). The zeta potential was measured at pH 7 using 2 mM KCl solution. To improve the data accuracy, each sample measurement was repeated 3 times. The membrane sample (4 × 5 mm size) was attached to the sample holder with double-sided tape to affix the membrane and make it flat. Several measurements were taken at different distances to the membrane surface according to the measuring device settings, with the resultant surface zeta potential obtained based on the abovementioned measurements.

##### Differential Scanning Calorimetry (DSC)

The significance of the hydration layer as a protective barrier against blood constituents and polymers was established in our previous research [49]. To assess the hydration state of the polymers, we employed DSC (differential scanning calorimetry). For this purpose, the membrane samples were prepared in both unmodified and modified forms and cut to a suitable size to fit in the DSC pan. To determine the equilibrium water content (*EWC*), the membranes were cut into specific dimensions. These samples were then immersed in deionized water at 30 °C for a duration of 24 h. The initial weight of the wet samples was measured as *W*_1_. Subsequently, the samples were dried in an oven at 75 °C for 24 h, and their weights were measured as *W*_2_. The *EWC* was calculated using Equation (1), as described previously.

For the DSC experiment, we utilized Q2000 TA instruments, which offer a temperature precision of ±0.1 °C. The hydrated samples were placed in aluminum pans, which were then sealed using an auto sealer. The pans were gradually cooled to −60 °C at a rate of 5 °C/min and kept at −60 °C for 5 min. Subsequently, the samples underwent a heating cycle from ice to water, at the same rate, until reaching 40 °C. The DSC heating profile of PES (polyethersulfone) was used as a control. By utilizing the DSC device, we quantified the amount of free water present. Additionally, based on the equations introduced in [50,51], we calculated the amount of stable water (non-freezable water) on the surface using Equation (2).

(1) $$EWC=\frac{w_{1}-w_{2}}{w_{1}}\times 100$$

(2) $$\begin{array}{c} EWC=\omega_{freezable}+\omega_{non-freezable}\\ =\frac{\Delta H_{freezable}}{\Delta H_{Bulk}}\times 100+\omega_{non-freezable} \end{array}$$

where $\Delta H_{Bulk}$ is equal to 355 J/g and $\Delta H_{freezable}$ can be obtained from the thermograms of the DSC.

#### 2.1.5. In Vitro Assessment of Inflammatory Biomarkers

Uremic blood from St. Paul Hospital patients was incubated in vitro with the membranes. The membrane specimens were incubated in uremic blood samples of CKD patients to assess the release of inflammatory biomarkers. A cohort of seven HD patients and two healthy controls from St. Paul’s Hospital dialysis center (Saskatoon, SK) were recruited. The blood samples were collected from participants following ethical approval of the study. Approximately 200 μL of serum was incubated at 37 °C in Eppendorf tubes (n = 7). After 30 min, the membrane was transferred to a separate clean tube. Next, 1 μL aliquot of the corresponding serum sample was prepared and subjected to Luminex assays (R&D Systems, a biotech brand, Minneapolis, MN, USA) using a Bio-Plex-200 (Bio-Rad, Hercules, CA, USA). The human magnetic Luminex assays used in this study are C5a, IL-1α, IL-1β, IL-6, vWF, and C5b-9. For the accuracy of the data, all the samples and controls were tested three times. A Shapiro–Wilk normality test was used for the statical analysis. The detailed information of these analyses was reported in our previous studies [8,10]. These data are also in agreement with the biomarkers models developed by Abdelrasoul et al. [49].

#### 2.1.6. Heparin Coating Stability Assessment

To assess the heparin coating’s stability, water filtration was performed. Water was pumped through the membrane at a flowrate of 200 mL/min for various amounts of time: 1 h, 2 h, and 4 h. After filtration, the membranes were dried and analyzed for surface charge.

## 3. Results and Discussion

### 3.1. Membrane Surface Modification

**Figure 4:**
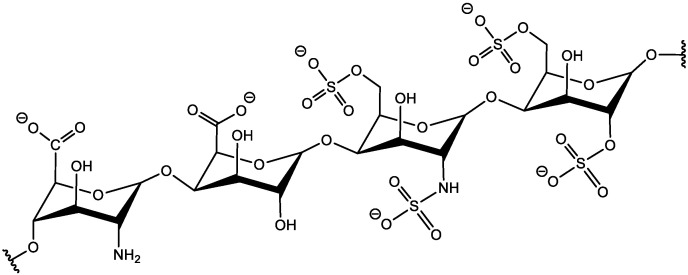
Chemical structure of heparin.

The heparin molecule (see Figure 4) has sulfonic and carboxylic groups that are negatively charged. Although heparin also contains NH_2_ groups that can be charged positively, the number of these groups is less than the carboxylic and sulfonic groups, thereby making the heparin total charge negative. This provides an opportunity to bind the heparin molecule via an electrostatic interaction with a positively charged surface and positively charged molecules. On the other hand, the presence of NH_2_ and OH groups can be used for the covalent immobilization of the heparin molecule.

**Figure 5:**
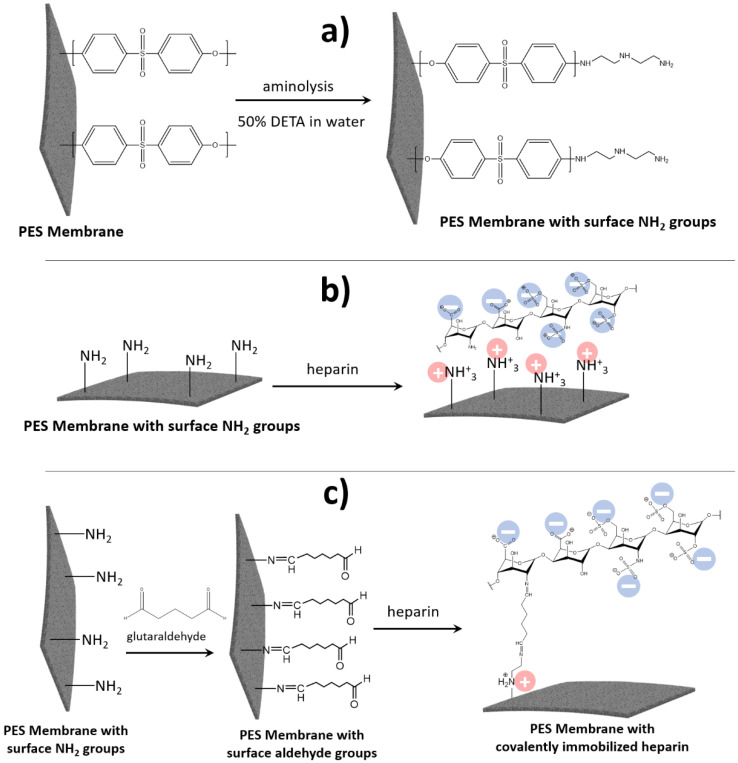
Scheme of PES modification with (**a**) surface NH_2_ groups; (**b**) immobilized heparin via pseudo-ZW complex; and (**c**) covalently attached heparin.

In this study, we compared the performance of PES membranes modified with heparin that was attached using both methods, i.e., covalently and via an electrostatic interaction (pseudo-ZW complex) (see Figure 5).

**Figure 6:**
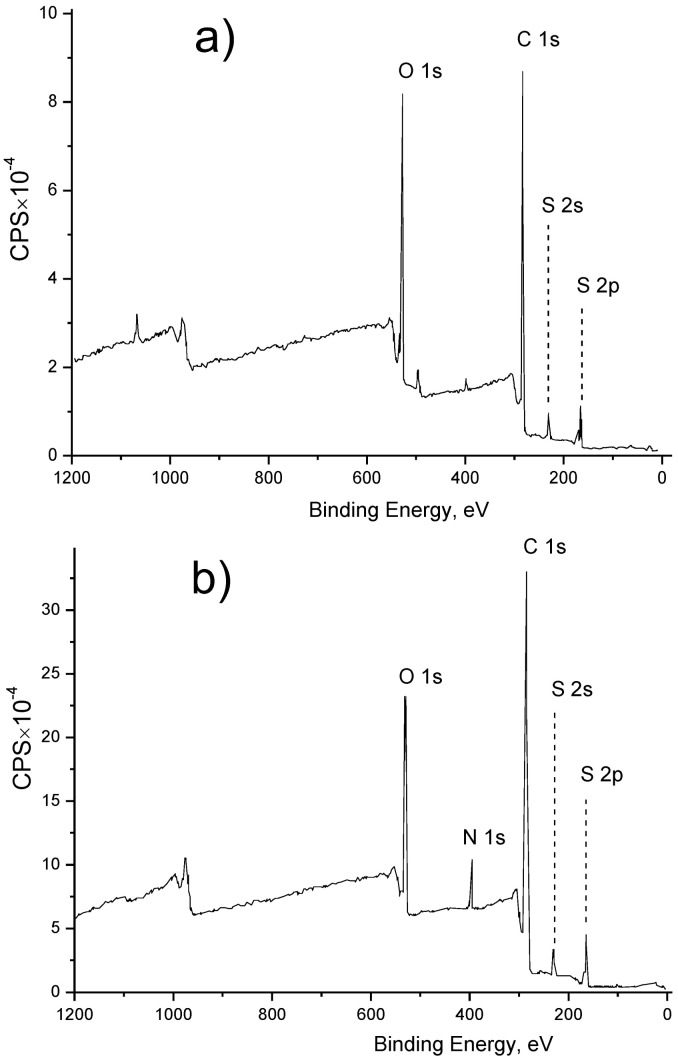

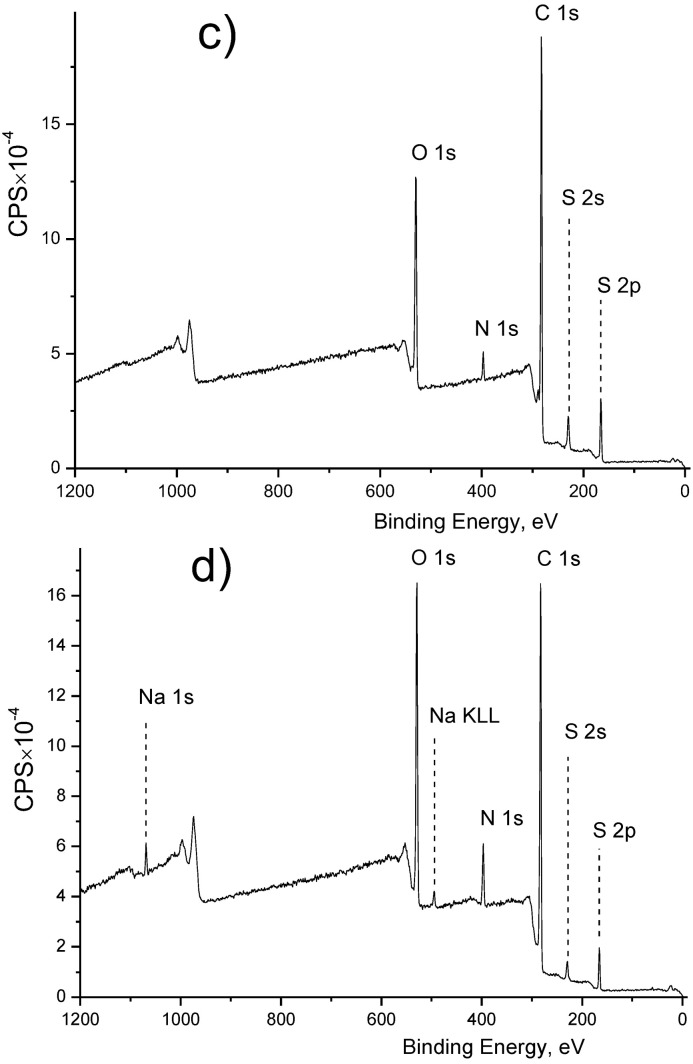
XPS spectra of PES membranes: (**a**) unmodified; (**b**) after aminolysis; (**c**) heparin–pseudo-ZW complex; (**d**) covalently attached heparin.

In the first step, we performed PES surface modification with NH_2_ groups via an aminolysis reaction (see Figure 5a). The presence of NH_2_ groups in the resultant membrane surface was confirmed with an XPS analysis (see Figure 6), revealing the presence of a nitrogen peak at 400 eV (see Figure 6b), which is nearly absent in the unmodified PES (see Figure 6a) and can be explained by the presence of a small amount of adsorbed N_2_ from the air on the PES surface. Then, the obtained NH_2_-contatining PES membrane was used for heparin immobilization using a different approach. For creating the pseudo-ZW complex, heparin was added to the NH_2_-containing PES membrane, resulting in the appearance of positive charges on the NH_2_ groups located on the PES membrane surface and negative charges on the heparin molecule (see Figure 5b). The amount of heparin immobilized by this approach should depend on the amount of NH_2_ surface groups, resulting in a total near-zero charge of the pseudo-ZW complex. This assumption was confirmed by measuring the surface zeta potential of the resultant membranes. Thus, the PES membrane with heparin immobilized via a pseudo-ZW complex possessed a reduced negative charge (−13 mV) compared with the unmodified PES membrane (−60 mV).

To immobilize heparin via covalent bonds, the NH_2_-surface-containing PES membrane was treated first with glutaraldehyde to create surface aldehyde groups that are able to react with NH_2_ groups present in heparin (see Figure 5c). This attachment also resulted in obtaining a PES membrane with a reduced negative charge (−9.16 mV), which can be explained by the presence of the NH group in the DETA molecule that does not react with GA, but can be charged positively. In addition, when the PES membrane is modified with covalently attached heparin, sodium (Na^+^) peaks (495 eV and 1070 eV) appear on the XPS spectra (see Figure 6d), but are absent on the XPS spectra for the aminated (Figure 6b) and heparin–pseudo-ZW-covered (Figure 6c) PES membranes. Sodium ions appear from heparin since heparin sodium salt was used for the surface modification (see Section 2). When heparin is attached covalently, its negative charge is also compensated with Na^+^ ions in addition to the NH_2_^+^ groups from the DETA molecules. When heparin is immobilized with a pseudo-ZW complex, the Na^+^ ions are substituted with charged amino groups. A similar phenomenon occurs when the PES surface is treated with DETA, which results in eliminating the Na^+^ ions that were present in the initial PES membrane due to the negatively charged sulfonic groups (see Figure 6a).

### 3.2. Enhancement of Membrane Surface Roughness

Besides the surface chemistry and charge, another important characteristic of the HD membrane is the surface roughness. When blood flows along the membrane’s surface, it is very important to avoid high turbulence caused by membrane rough surface because it results in increased shear stress, which, in turn, provokes red blood cell (RBC) rupture [8,49]. RBC rupture, or hemolysis, is a highly undesirable phenomenon for HD patients that triggers protein adsorption, platelet adhesion, and further coagulation reactions that result in thrombus formation and many clinical symptoms ranging from headaches, back pain, and hypertension to death [50].

**Figure 7:**
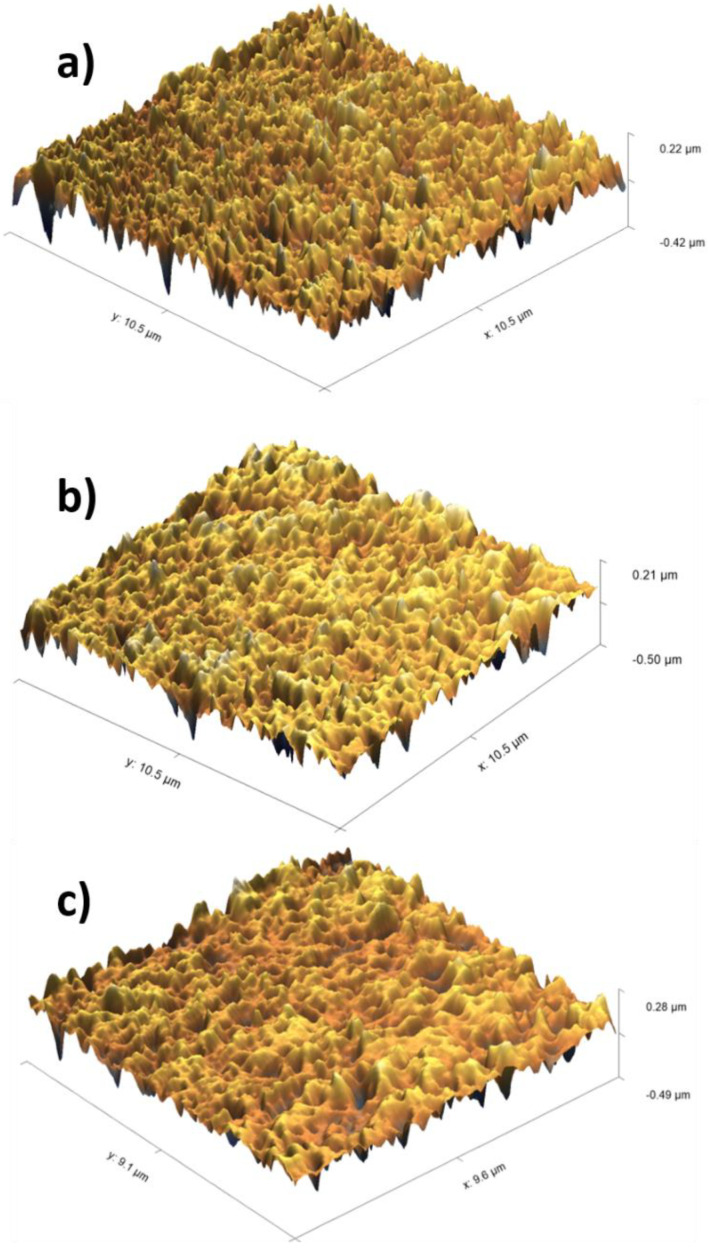
AFM images of PES membranes: (**a**) unmodified membrane; (**b**) covalently attached heparin; (**c**) heparin attached via pseudo-ZW complex.

The obtained membranes were analyzed using AFM and compared to the unmodified membrane (see Figure 7). The unmodified PES membrane (Figure 7a) appeared rougher compared to the modified PES membranes (Figure 7b,c), which could be considered as additional evidence of heparin immobilization on the PES surface. When the different methods of heparin immobilization were compared, heparin being immobilized via an electrostatic interaction appeared to result in a smoother surface, though the height of the membrane roughness increased from 220 nm for the unmodified PES membrane to 280 nm for the heparin–pseudo-ZW-coated PES membrane.

It is also worth mentioning that immobilization of heparin via both methods does not appear to significantly block the membrane pores; it is very important to consider the membrane permeability and performance during the experiments.

### 3.3. Assessment of FB Depletion

Protein loss during HD sessions is the price that is paid for removing protein-bound uremic toxins (PBUT). PBUT, such as 3-carboxy-4-methyl-5-propyl-2-furanpropionic acid, binds to albumin by 98% and its presence in blood inhibits erythropoiesis [51]. On the other hand, p-cresyl and indoxyl sulfates bind to albumin at nearly 100% and cause endothelial proliferation and wound repair inhibition [52]. Low albumin concentrations that were observed after HD sessions were reported to be associated with high mortality [53,54]. On the other hand, a moderate loss of albumin during an HD session does not pose a high risk for HD patients. Moreover, healthy organisms eliminate about 1.3 g of HSA per day by kidney filtration [55], and the majority of HD patients are able to increase their HSA synthesis rate to sustain normal HSA concentration in the blood [56].

Another important human serum protein is FB, which is present in blood at a much lower concentration (2–4 mg/mL) than has. It is a critical protein that should be present in the blood at a concentration no less than 1 mg/mL to sustain hemostasis [57]. Moreover, severe bleeding can be anticipated in patients with plasma fibrinogen levels below 0.5–1 mg/mL. Thus, in our research, we have focused on FB loss during ultrafiltration.

**Figure 8:**
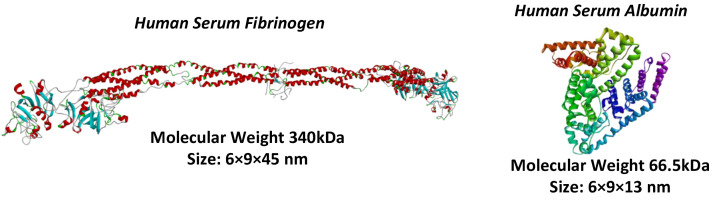
Dimension comparison of FB (**left**) and HSA (**right**).

The difference between FB and HSA proteins is worth mentioning (see Figure 8). FB has a rod-like structure, whereas HSA is more similar to a globular shape. Despite FB having a large linear size (45 nm), it is still able to pass through the membrane pores due to its two other small dimensions that are comparable to HSA’s size, although a reduced capability of FB to penetrate the HD membrane is expected.

**Figure 9:**
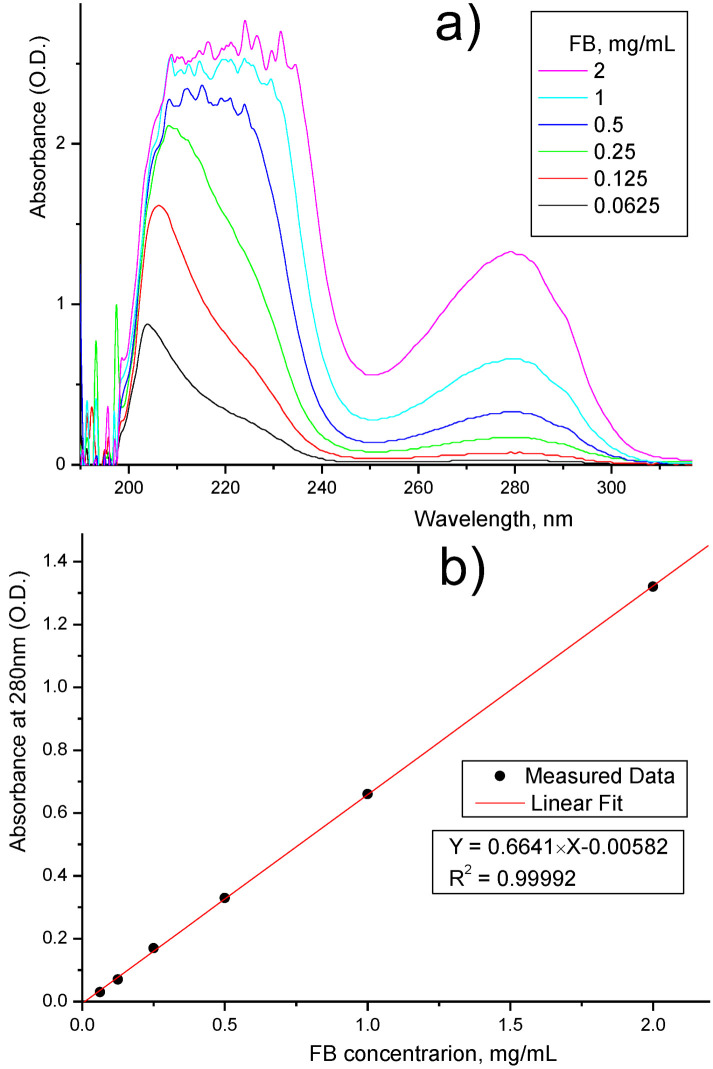

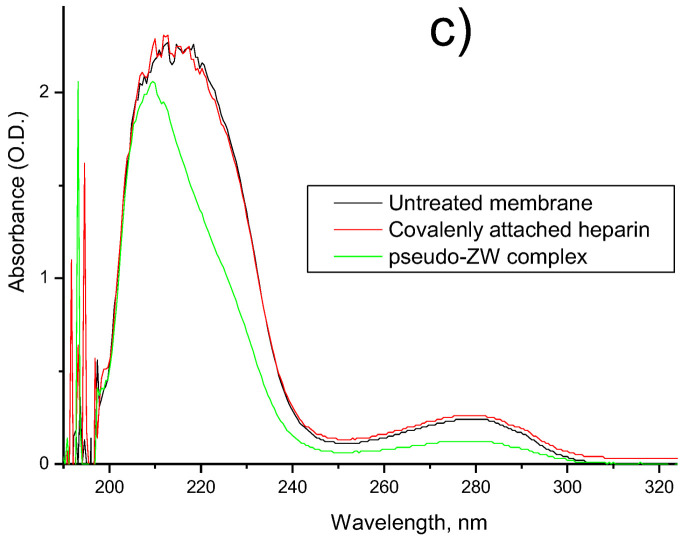
UV data for FB permeation test: (**a**) calibration curves for FB; (**b**) calibration equation; (**c**) UV spectra of collected permeates.

To investigate FB loss through the dialysis membranes, we used an FB solution (2 mg/mL in saline) and measured the concentration of FB in the permeate using UV analysis (see Figure 9).

First, we prepared six FB solutions with known concentrations and collected spectra for these solutions (see Figure 9a). The characteristic UV adsorption of FB was observed at 280 nm wavelength, for which we plotted the calibration data (see Figure 9b), which can be quite accurate when approximated with a straight line (R^2^ = 0.99992). Moreover, the interception with the Y-axis was only 0.00582 (O.D.), which means that the obtained equation can be used to precisely detect and estimate even small FB concentrations. Then, we collected the UV spectra for the permeates (see Figure 9c) and by using the abovementioned calibration data, we calculated the FB concentration in the filtrate and FB retention. The results are presented in Table 1. The ultrafiltration coefficient of the membrane was around 50 mL/h·mmHg, and it was not been affected after coating the membrane.

The covalent immobilization of heparin on the PES surface does not significantly change the retention (79.8% vs. 81.3% for the unmodified PES membrane) of FB; moreover, some increase of the FB concentration in the permeate was observed for the PES membrane with covalently attached heparin. When heparin was attached to the PES membrane via an electrostatic interaction (i.e., pseudo-ZW complex), a significant improvement in the FB retention (90.5%) was detected. 

### 3.4. Assessment of In Vitro FB Adsorption on Membrane Surface

**Figure 10:**
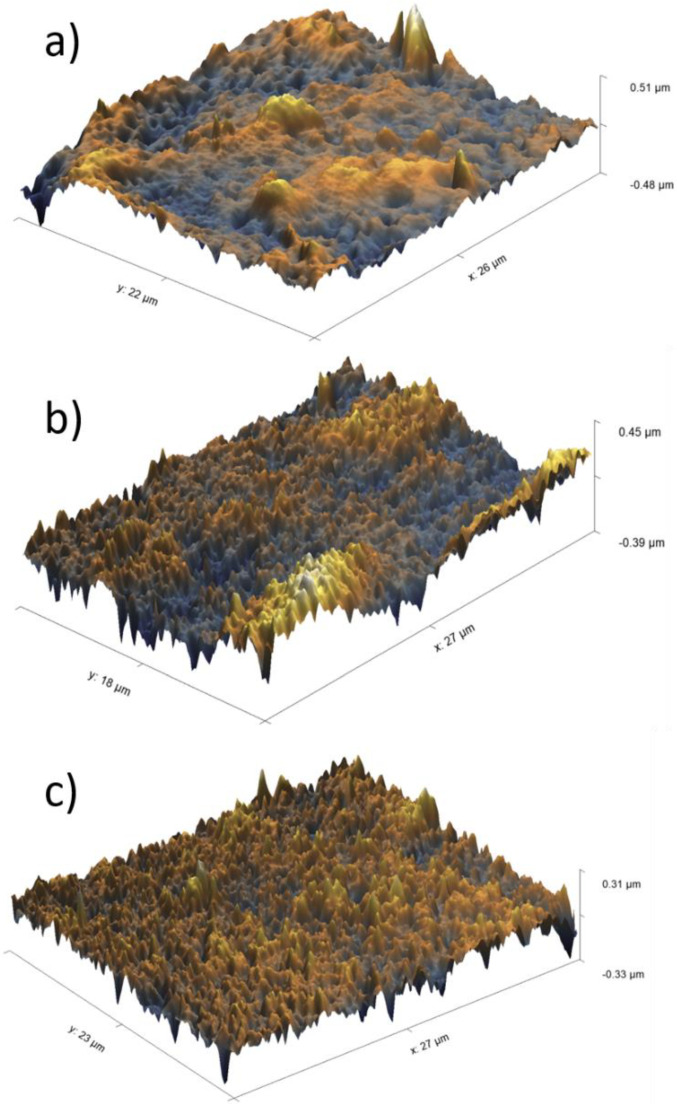
AFM images of FB thickness on membrane surface: (**a**) unmodified membrane; (**b**) covalently attached heparin; (**c**) heparin attached via pseudo-ZW complex.

Protein adsorption in the hemodialysis membrane is undesirable and reduces the treatment effectiveness due to poor clearance; it can also lead to a series of side effects and long-term implications that can compromise patients’ quality of life [9]. Figure 10 depicts the FB adsorption on the unmodified and modified membranes’ surfaces. FB had a thickness of 51 µm, 45 µm, and 31 µm on the unmodified PES membrane, covalently attached heparin, and heparin–pseudo-ZW complex, respectively. It is worth mentioning that the average measurement of the membrane roughness was 0.22 µm, 0.21 µm, and 0.28 µm for the unmodified PES membrane, covalently attached heparin, and heparin–pseudo-ZW complex, respectively. The results prove that the heparin–pseudo-ZW complex had the minimum affinity towards FB adsorption and the minimum amount of FB adsorbed on the membrane surface. Consequently, reduced concentrations of inflammatory biomarkers were released in the patients’ serum.

### 3.5. In Situ X-ray Synchrotron SR-μCT Imaging for Layer-by-Layer FB Adsorption Assessment

Blood clotting is a highly undesirable process that causes severe health problems and even death for HD patients. This process is believed to start with protein adsorption on the HD membrane surface. Fibrinogen plays a major role in this process since it substitutes other proteins due to the Vroman effect and participates in further platelet adhesion and their activation.

**Figure 11:**
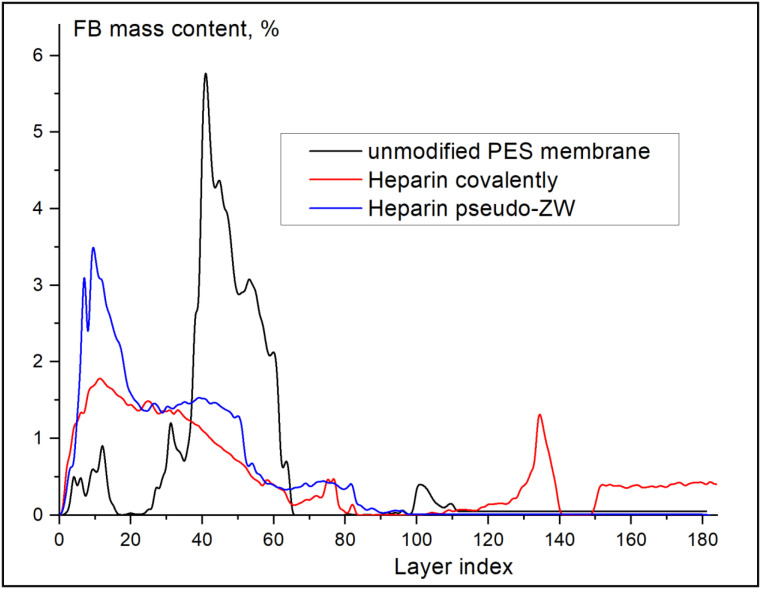
FB distribution across membranes’ thickness for unmodified and modified PES membranes according to SR-μCT analysis.

Although covalently attached heparin does not enhance FB retention, it does result in a dramatic change in FB distribution across the membrane thickness (see Figure 11). As shown in Figure 11, the unmodified PES membrane held the majority (nearly 91%) of the FB inside the membrane in the low and middle (layer index from 20 to 75) areas, which negatively affects the HD performance since the adsorbed FB can provoke blood coagulation and clotting. When heparin was attached covalently to the PES membrane, the majority (nearly 55%) of FB was located on the membrane surface (0 to 40 index layers) and could be removed by the bloodstream. The remainder of the FB was located in the membrane’s bottom area (layer index 120 to 140). 

**Figure 12:**
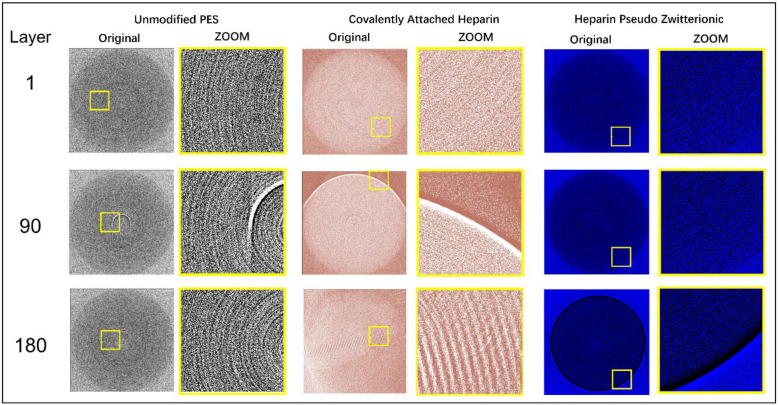
CT slices of membrane layers (**top**, **middle**, and **bottom**).

On the other hand, when heparin was immobilized via the pseudo-ZW complex, the amount of FB located at the membrane surface (0 to 20 index layers) increased almost twice (from 26% to 40%) compared with the covalently attached heparin, and no FB was found at the high index layers. Thus, heparin attached to the PES membrane surface via an electrostatic interaction (pseudo-ZW complex) is preferred. Figure 12 presents the CT scans of the membrane layers (top, middle, and bottom) for each sample imaged using synchrotron radiation; the CT scans were used to obtain the quantitative data presented in Figure 11.

### 3.6. Assessment of Membranes’ Non-Freezable Water

**Figure 13:**
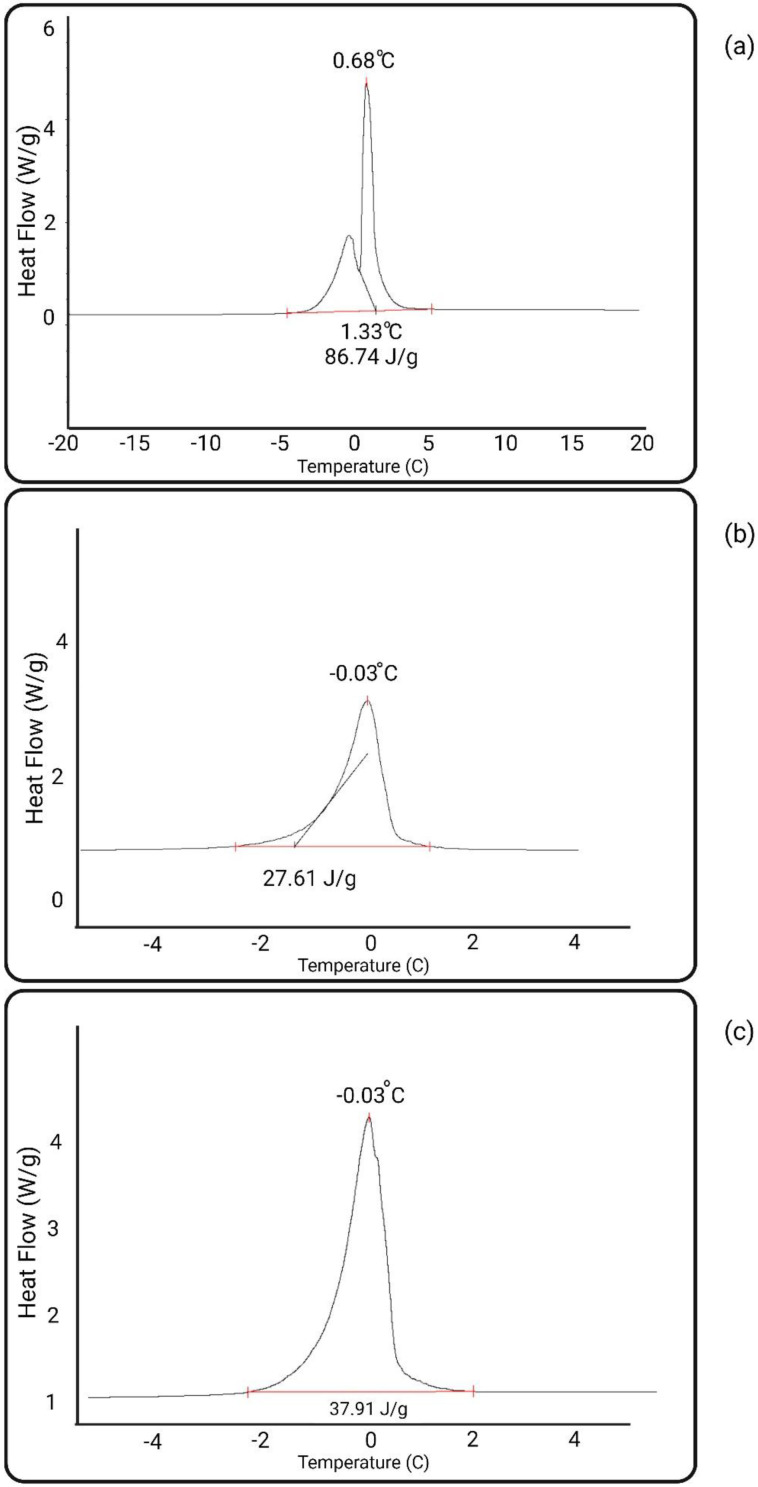
DSC peak of neat and modified membranes: (**a**) neat PES, (**b**) heparin–pZW, (**c**) PES-covalent heparin; temperature precision ±0.1 °C.

Figure 13 shows the DSC scans of the neat PES and modified membranes. Table 2 summarizes the non-freezable water content and freezing point of the membranes.

Both heparin-coated PES membranes demonstrated a significant increase in the non-freezable water content (30–32%) compared with the unmodified PES (2.84%). The increase in the amount of strongly immobilized (non-freezable) water resulted from the zwitterionic structure of both heparin-covered PES membranes (see Figure 5). It should be noted, however, that the amount of non-freezable water for the heparin immobilized via an electrostatic interaction was slightly higher than that for the covalently immobilized heparin. The slightly lower content of non-freezable water in the covalently attached heparin could possibly be explained by the hindrance of NH_2_^+^ charges by the GA molecules.

### 3.7. Ex Vivo Study of Inflammatory Biomarkers Released in Patients’ Blood

**Figure 14:**
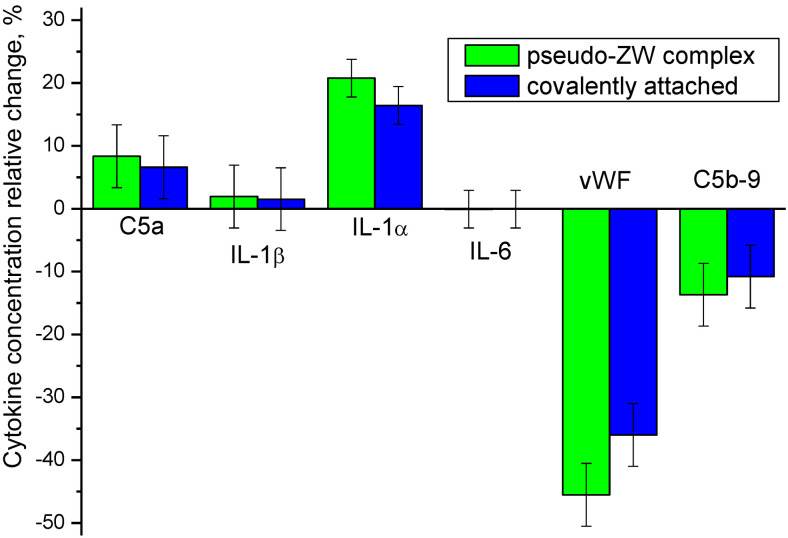
Relative change in cytokine concentration for PES modified membranes as compared to unmodified PES membrane.

To investigate the biocompatibility of the modified PES membranes, we performed an in vitro test with the uremic serum of a patient group to study the change in the cytokine concentration when the biomaterial contacted the PES membrane surface. The results of the relative change in cytokine concentrations compared to the unmodified PES membrane are shown on Figure 14. The absolute concentrations of the cytokines are given in Table 3.

#### 3.7.1. C5a

Proteins’ adsorption tendency to dialysis membranes is thought to play a role in the release of inflammatory cytokines and vWF [58,59]. Human C5a anaphylatoxin is a bioactive biomarker that owns both spasmogenic and leukocyte-related characteristics. Various properties of C5a make it a critical component for the normal host defense mechanism of the human body. However, the increased level of the biomarker in dialysis sessions could promote the well-known complications of hemodialysis [60]. It has been proven that the generation of the complement anaphylatoxin C5a could lead to the expression of active tissue factor (TF) in ESRD. This will contribute to hemodialysis-induced thrombogenesis [61]. The modified PES membranes showed a C5a level higher by 6–8% compared with the unmodified PES membrane.

The increased level of C5a for the zwitterionized membranes here could be due to more negatively charged surfaces. 

#### 3.7.2. Interleukins

Cytokines, such as interleukin-1β (IL-1β) and IL-6, may induce an inflammatory state and are believed to play a significant role in dialysis-related morbidity [62]. It has been proven that the basal expression of IL-6 and TNF-α is not a function of the membrane’s hemocompatibility, since they are more affected by the endotoxin content of the dialysate [63]. We did not observe any noticeable change in the IL-6 concentration for the modified PES membranes, although IL-1α increased by 17–21% for the modified PES membranes. IL-1β demonstrated only a 1.5–2% increase when the PES membrane was covered with heparin.

#### 3.7.3. vWF

vWF is a glycoprotein that takes part in hemostasis and is an identifier for endothelial cell stimulation [64]. Based on the literature, a pronounced inflammatory level of cytokines is thought to affect the increase of vWF [65]. We observed significantly lower concentrations of vWF for both of the modified PES membranes. Thus, heparin immobilization via the pseudo-ZW complex resulted in a 46% decrease of this cytokine level, while the covalently attached heparin reduced its concentration by 36%.

vWF is either created in endothelial structures and megakaryocytes or through the granules of platelets [66]. Accordingly, vWF is a known predictor of cardiovascular shock [67] due to its “complement thrombogenesis-linking nature”. The correlation between the adsorption of plasma proteins, such as fibrinogen, to the membrane and the amount of vWF has been mentioned elsewhere [68]. 

#### 3.7.4. C5b9

Our measurements reflected a lower level of C5b-9 for the modified membrane as compared to the pristine PES membrane. The reduction in the C5b-9 concentration was measured to be 14% for the zwitterionized PES membrane and 11% for the covalently attached heparin. The plasma terminal C complex, the C5b-9 complex, is a stable and reliable marker of biocompatibility. Measuring the concentration of this cytokine could reflect the hemocompatibility extent of the membrane in regard to the complement cascade [69]. The enhanced performance (lower production of C5b-9) is due to the limited activation of the complement cascade. Another potential property of the heparin-modified membrane that will support a lower level of C5b-9 production is the stable hydration layer. Since a protected water layer is formed on the PES-heparin membrane, the attachment of macromolecules as the initiation mechanism of C5b-9 is prohibited. 

Compared with the unmodified PES membrane, both heparin-modified membranes resulted in an increase of C5a, IL-1α, and IL-1β cytokines, whereas the pseudo-ZW complex caused a slightly increased release of cytokines as compared with the covalently attached heparin, especially when IL-1α is considered. No noticeable change in the IL-6 concentration was observed. The concentration values of both vWF and C5b-9 significantly dropped when the heparin-modified PES membranes were used. It should be pointed out that heparin immobilized via a pseudo-ZW complex results in a larger reduction of the cytokine concentration in comparison with the covalently attached heparin by 10% for vWF and 3% for C5b-9. The most noticeable change was observed for vWF (von Willebrand factor). This glycoprotein is involved in the platelet adhesion process and an increase of its concentration is associated with a high risk of thrombosis [70]. Thus, heparin immobilized via a pseudo-ZW complex is expected to reduce the risk of blood clotting.

### 3.8. Assessment of the Stability of Membrane Coatings

In this study, we evaluated the stability of heparin-coated membranes by assessing their surface charge over a filtration period of up to 4 h. The unmodified PES membrane exhibited a surface charge of −60 mV. The surface charge measurements presented in Table 4 demonstrate that the heparin coatings remained relatively stable during the 4 h filtration, as they did not revert to the −60 mV level observed in the unmodified PES membrane.

While these results suggest promising initial stability, we acknowledge that long-term investigations are essential to further validate and optimize the coating’s performance. Our ongoing research is dedicated to addressing this aspect by conducting extended stability studies beyond the initial 4 h duration. By carefully monitoring the surface charge over an extended period, we aim to identify potential changes and fluctuations, ensuring the development of more robust and long-lasting heparin-coated membranes.

Based on the data in Table 4, it can be concluded that both types of heparin coatings are stable for 4 h. 

## 4. Conclusions

The PES membrane was modified with heparin using two different approaches. The first approach involved the electrostatic interaction between negatively charged heparin macromolecules and the positively charged PES surface, resulting in the formation of a pseudo-zwitterionic (pZW) complex. The second approach was the covalent attachment of heparin molecules to the PES surface. In both cases, the modified membranes exhibited a reduced negative charge (around −10 mV) compared to the unmodified PES membrane (−60 mV). The surface of both modified membranes appeared smoother, as detected by AFM analysis.

No significant influence on fibrinogen (FB) retention was observed for the heparin covalently coated PES membrane, as both the unmodified and heparin-coated membranes retained approximately 80% of FB. However, the PES membrane immobilized with heparin via the pZW complex showed a significant improvement in FB retention (90.5%). Furthermore, the distribution of FB across the membrane thickness was shifted to a greater extent toward the membrane injection side for the pZW complex, indicating reduced membrane fouling by this protein.

Both heparin-coated membranes exhibited a significant improvement in the tightly bound water content (around 30%) compared to the unmodified membrane (2.84%), as revealed by DSC analysis. This improvement in the water content contributes to better biocompatibility. The heparin attached via the pZW complex showed a slightly higher content of strongly bound water.

The clinical study demonstrated an improvement in the hemocompatibility for both heparin-coated membranes, although the pZW complex exhibited better performance in terms of reducing the von Willebrand factor (46% for the pZW complex versus 36% for covalently attached heparin) and C5b-9 cytokine release (14% for the pZW complex versus 11% for covalently attached heparin).

## Figures and Tables

**Table 1:** The permeability of PES membranes for FB based on UV analysis.

Membrane	FB Concentration in Injected Solution, mg/mL	FB Concentration in Permeate (Based on UV Analysis), mg/mL	FB Retention, %
Unmodified PES	2	0.374	81.3
Covalently attached heparin		0.405	79.8
Heparin–pseudo-ZW		0.190	90.5

**Table 2:** Percentage of stable water for PES neat and modified membranes; temperature precision ±0.1 °C.

Membrane	Non-Freezable Water (%)	Freezing Point (°C)
PES	2.84	0.68
PES Heparin–pZW	32.22	−0.03
PES Covalent Heparin	30.99	−0.03

**Table 3:** Absolute concentrations of cytokines in in vitro experiments for PES membranes.

Membrane	Cytokine Concentration, pg/mL					
	C5a	IL-1β	IL-1α	IL-6	vWF	C5b-9
Unmodified PES	139,502	4.861	5.278	12.653	497	3806
Heparin attached covalently	148,726	4.936	6.146	12.645	318	3395
Heparin–pseudo-ZW	151,169	4.956	6.376	12.643	270	3286

**Table 4:** PES membranes surface charge variation during water filtration.

Filtration Time, Hours	PES Heparin–pZW	PES Heparin Covalently
0	−13 ± 2.66	−9.16 ± 2.18
1	−7.59 ± 0.448	−5.69 ± 1.57
2	−11.2 ± 0.647	−8.84 ± 1.56
4	5.66 ± 1.30	5.09 ± 1.21

## Data Availability

The raw/processed data required to reproduce these findings cannot be shared at this time, as the data is critical to the ongoing research.
