# Correction: Kalugin et al. Heparin-Immobilized Polyethersulfone for Hemocompatibility Enhancement of Dialysis Membrane: In Situ Synchrotron Imaging, Experimental, and Ex Vivo Studies. *Membranes* 2023, *13*, 718

**DOI:** 10.3390/membranes16030102

**Published:** 2026-03-11

**Authors:** Denis Kalugin, Jumanah Bahig, Ahmed Shoker, Amira Abdelrasoul

**Affiliations:** 1 Department of Chemical and Biological Engineering, University of Saskatchewan, 57 Campus Drive, Saskatoon, SK S7N 5A9, Canada; 2 Division of Biomedical Engineering, University of Saskatchewan, 57 Campus Drive, Saskatoon, SK S7N 5A9, Canada; 3 Kinesiology, University of Saskatchewan, 87 Campus Drive, Saskatoon, SK S7N 5B, Canada; 4 Nephrology Division, College of Medicine, University of Saskatchewan, 107 Wiggins Rd., Saskatoon, SK S7N 5E5, Canada; 5 Saskatchewan Transplant Program, St. Paul’s Hospital, 1702 20th Street West, Saskatoon, SK S7M 0Z9, Canada

## Text Correction

In the original publication [1], the citation of new reference [47] was not included in Figure 3, the schematic diagram illustrating the software functionality and the standard image analysis workflow in ImageJ software. The citation has now been inserted in the Materials and Methods Section 2.1.2. and in the Figure 3 caption as reference [47]. The figure was adapted from previously published literature with appropriate permission. The new reference [47] and updated caption appears below:

47. Rueda Plá, G.; Maghami, M.; Doan, H.; Zhu, N.; Abdelrasoul, A. Investigation on the morphology and the permeability of biomimetic cellulose triacetate (CTA) impregnated membranes (IM): In-situ synchrotron imaging, experimental and computational studies. *Mater. Chem. Phys.* **2022**, *292*, 126755.

**Figure 3.** (**a**) Raw image of CT scans and (**b**) zoomed image of selected area. Adapted from [47].

With this correction, the order of some references has been adjusted accordingly.

## Addition of Institutional Review Board Statement Details

To increase specificity and transparency of the study [1], the Institutional Review Board approval number and date have been added to the end of the Institutional Review Board Statement as follows: “..., approval number BIO 1190, issued on 7 June 2019.”

The authors state that the scientifsic conclusions are unaffected. This correction was approved by the Academic Editor. The original publication has also been updated.
